# Preparation, Structure, Function, and Application of Dietary Polysaccharides from *Polygonatum sibiricum* in the Food Industry: A Review

**DOI:** 10.3390/molecules30051098

**Published:** 2025-02-27

**Authors:** Peilin Li, Huimin Yao, Hao Yue, Jiali Huang, Qi Wang, Chuanbo Ding, Lina Ma, Xinglong Liu, Min Yang

**Affiliations:** 1 College of Traditional Chinese Medicine, Jilin Agriculture Science and Technology College, Jilin 132101, China; 17781715552@163.com (P.L.); huiminyao0921@163.com (H.Y.); 19141317361@163.com (J.H.); chuanboding0506@163.com (C.D.); malina@jlnku.edu.cn (L.M.); 2 The Key Laboratory of Utilization and Protection of Animal and Plant Resources in Changbai Mountain, Jilin 132101, China; 3 College of Health Management, Changchun University of Traditional Chinese Medicine, Changchun 130117, China; 18844142914@163.com; 4 College of Food Science and Engineering, Changchun University, Changchun 130022, China; 15843018487@163.com

**Keywords:** *Polygonatum sibiricum* polysaccharides, preparation process, structural analysis, biological activity, functional food

## Abstract

*Polygonatum sibiricum* is one of the most widely used plants in the Liliaceae family, renowned for its dual medicinal and edible properties. *Polygonatum sibiricum* polysaccharides, as the main pharmacological active ingredient of *Polygonatum sibiricum*, have various excellent physiological activities, such as antioxidant, immune enhancement, and hypoglycemic activities. Through extraction, purification, and structural analysis, the influence and mechanism of the molecular weight and glycosidic bonds of *Polygonatum sibiricum* polysaccharides on the pharmacological effects, as well as their structure–activity relationship, can be explored in more detail. With the increasing demand for *Polygonatum sibiricum* polysaccharide products, *Polygonatum sibiricum* has been widely used in the fields of medicine, food, and biochemistry, and various green and harmless products containing *Polygonatum sibiricum* polysaccharides have been developed for different populations. This study summarizes the extraction, structure, and function of *Polygonatum sibiricum* polysaccharides, and it further explores their applications in the food industry, including in beverages, health foods, additives, and food packaging. Overall, *Polygonatum sibiricum* polysaccharides have been proven to be a promising natural product and have been introduced into the food system. It is worth mentioning that further efforts and time are needed in the future to expand the deep processing of and feasibility research on *Polygonatum sibiricum* polysaccharides while exploring their bioactive molecular mechanisms in depth, laying the foundation for their product development and clinical applications.

## 1. Introduction

*Polygonatum sibiricum* is a plant species in the Liliaceae family belonging to the genus *Polygonatum sibiricum*. There are approximately 79 varieties in the world, including 39 species in China which are mainly distributed in the southwest, northwest, northeast, and central regions. *Polygonatum sibiricum*, first recorded as a superfine-grade medicinal herb in the “Record of Famous Doctors”, is known by the names “Chonglou”, “Jige”, “Luzhou”, and “Jiuqiong”. The record describes “*Polygonatum sibiricum*, sweet, flat and non-toxic”. In the Pharmacopoeia of the People’s Republic of China 2020, *Polygonatum kingianum*, *Polygonatum sibiricum*, and *Polygonatum cyrtonema* are the sources of *Polygonatum sibiricum*, which are traditional Chinese medicinal materials. Interestingly, *Polygonatum sibiricum* is called “Da Huang Jing”, “Jiang Xing Huangjing”, and “Ji Tou Huangjing” according to its different shapes.

In the list of medicinal and edible products released by the Ministry of Health of China in 2002, *Polygonatum sibiricum* is included due to its various health-promoting effects. *Polygonatum sibiricum* comprises various chemical components, including polysaccharides [1], steroidal saponins [2], flavonoids [3], alkaloids [4], cardiac glycosides, lignin, vitamins, and various amino acids [5]. The main pharmacological activities of *Polygonatum sibiricum* include anti-fatigue, hypoglycemic, immune enhancement, antioxidant, anti-aging, anti-tumor, etc., activities, making it widely used in the field of medicinal and edible homology.

For people currently pursuing a healthy life, the development of health care products with *Polygonatum sibiricum* as the main raw material can meet their various health pursuits. *Polygonatum sibiricum* is a category of plant with a medicinal and edible homology which has a unique flavor and extraordinary value. Especially, *Polygonatum sibiricum* has a variety of pharmacological activities and can be used to treat multiple clinical diseases. Therefore, it is very popular on the health product market, with a variety of product forms, including powders, pills, tablets, etc. The functions of common products such as *Polygonatum sibiricum* rice wine and *Polygonatum sibiricum* oral liquid mainly include anti-fatigue, hypoglycemic, and immunity-enhancing effects. In addition, *Polygonatum sibiricum* is mostly used for clinical treatment in the form of traditional Chinese patent medicines, simple preparations, or decoctions, mainly for brain cognitive impairment, the recovery of brain memory functioning, diabetes, and other symptoms, such as Tangmaikang capsules. *Polygonatum sibiricum* also has anti-oxidation and anti-aging functions, and it contains active ingredients of beauty, and so cosmetic products based on *Polygonatum sibiricum* also have a development trend. Based on extensive research on *Polygonatum sibiricum* in recent years, the product development of *Polygonatum sibiricum* has received strong theoretical support. Moreover, in an increasingly strict environment, the source of *Polygonatum sibiricum* is strictly controlled, and the demand for related products has created positive opportunities for the research and development of *Polygonatum sibiricum*.

Natural products derived from plants have received significant attention and have wide application, especially in the food industry, due to their low toxicity and extensive biological activity. Compared with other plant components, the safety of polysaccharides has been widely recognized. Currently, polysaccharides are the main pharmacological active ingredient of *Polygonatum sibiricum*, which has various activities, especially hypoglycemic [6], immunomodulatory [7], antioxidant [8], anti-tumor [9], anti-osteoarthritis [10], antidepressant [11], etc., activities, and has been widely used in the fields of medicine, food, and biochemistry, in which it is playing an important role. Currently, it can be seen from the development trend of *Polygonatum sibiricum* that the research on dietary polysaccharides of *Polygonatum sibiricum* is relatively balanced both domestically and internationally, and the research and development of dietary polysaccharides of *Polygonatum sibiricum* is more extensive in China. Moreover, both domestically and abroad, the focus of investigations into *Polygonatum sibiricum* dietary polysaccharides is from different angles, including its chemical composition, content determination, biological activity, and pharmacological effects, which also explore its antioxidant, anti-diabetes, and anti-osteoporosis role and other aspects. Surprisingly, foreign research especially involves therapeutic effect and mechanism research on diseases, including anti-herpetic activity research [12], intestinal microorganism-related research [13], Alzheimer’s disease [14], diabetes with retinopathy [15], and cancer. Domestic research focuses more on its medicinal and food homology value [16], extraction method [17], and processing techniques [18] and also includes intervention in cell apoptosis, treating osteoporosis, and research on the mechanisms of liver and kidney injury.

**Figure 1:**
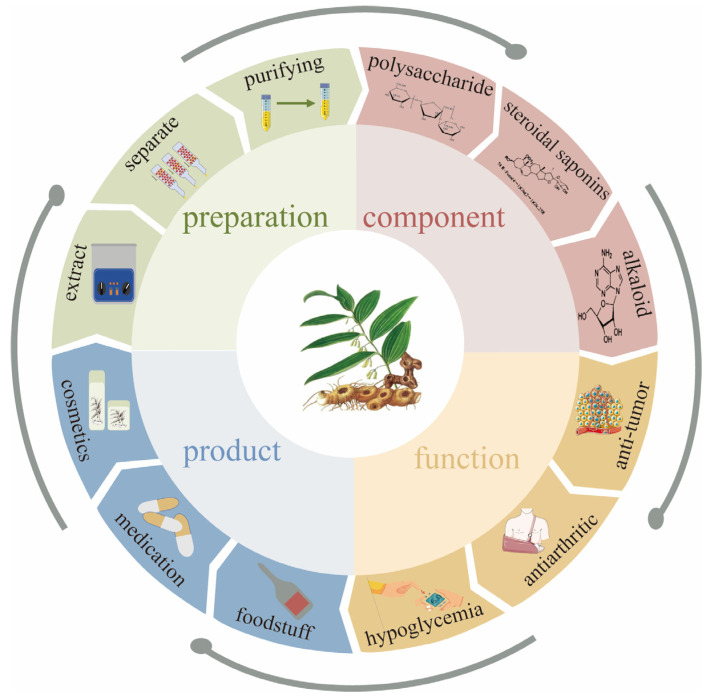
Preparation, ingredients, activities, and products of *Polygonatum sibiricum*.

This study provides a systematic analysis and summary of the preparation methods, structural characterization, pharmacological activities, and applications of *Polygonatum sibiricum* dietary polysaccharides in food and related fields, the aim of which is to provide a reference for the research on *Polygonatum sibiricum* dietary polysaccharides and to provide a more scientific and favorable basis for the rational utilization of *Polygonatum sibiricum* dietary polysaccharides and the development of their products in the future. The preparation, ingredients, activities, and products of *Polygonatum sibiricum* are shown in Figure 1.

## 2. Preparation Process of Dietary Polysaccharides from *Polygonatum sibiricum*

### 2.1. Study on Extraction Technology of Dietary Polysaccharides from Polygonatum sibiricum

**Figure 2:**
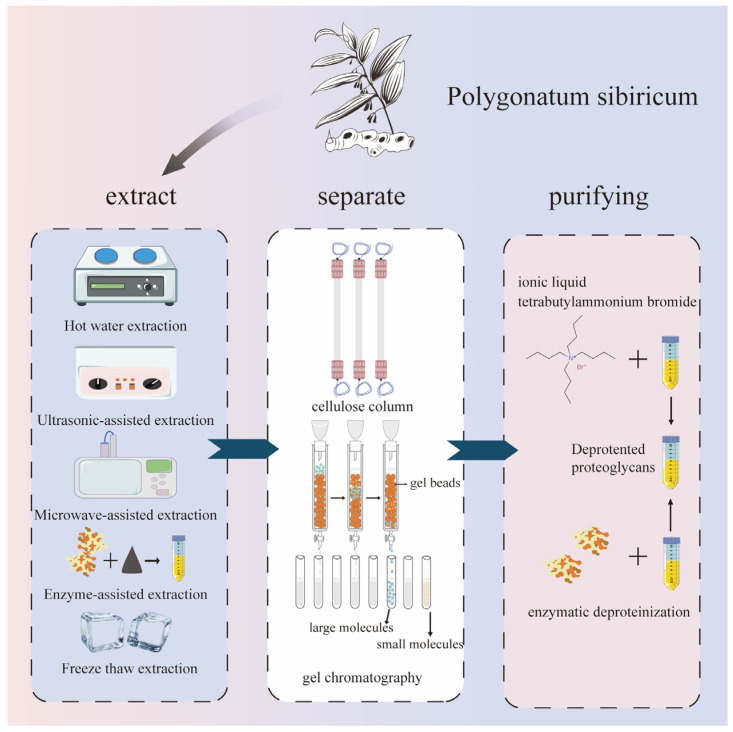
Extraction, separation, and purification of polysaccharides from *Polygonatum sibiricum*.

In most cases, it is easier to extract polysaccharide substances using hydrophilic solvents, and selecting the appropriate extraction methods is more conducive to the extraction efficiency. Currently, hot water extraction, ultrasound-assisted extraction, microwave-assisted extraction, enzyme-assisted extraction, ultra-high-pressure extraction, acid–base extraction, deep eutectic solvent extraction, and microbial extraction are mainly used. These methods are mostly environmentally friendly and pollution-free, and they can achieve the green extraction of *Polygonatum sibiricum* polysaccharides. The extraction, separation, and purification of polysaccharides from *Polygonatum sibiricum* are shown in Figure 2.

#### 2.1.1. Extraction of Polygonatum sibiricum Polysaccharides via the Physical Method

##### Ultrasonic-Assisted Extraction

In most cases, ultrasound-assisted extraction is more suitable in the laboratory as it can improve the extraction efficiency, shorten the extraction time, accelerate the extraction process, and utilize the cavitation effect to enhance the dissolution rate and effectiveness of raw materials.

Chen et al. used CO_2_-triggered switchable hydrophilic solvents based on different amines and water for the ultrasound-assisted extraction of dietary polysaccharides from *Polygonatum sibiricum* and studied the effects of the main influencing factors, such as the solid–liquid ratio, extraction time, extraction temperature, and ultrasound power through single-factor experiments. The results show that the maximum extraction rate of dietary polysaccharides from *Polygonatum sibiricum* is 399.2 mg/g when the extraction time is 60 min, the extraction temperature is 50 °C, the solid–liquid ratio is 1:20, and the ultrasonic power is 500 W [19]. Chen et al. used 1-ethyl-3-methylimidazolium bis[(trifluoromethyl)sulfonyl]imide ([EMIM] [Tf_2_N]) as a co-solvent and employed ionic liquid ultrasound-assisted hydrodistillation (IL-U-HD) to extract turmeric essential oil (CEO). Under the optimal process conditions of a water-to-liquid ratio of 43:1, a liquid-to-material ratio of 12:1, an ultrasound time of 37 min, and an ultrasound temperature of 50 °C, the average yield of CEO was 6.88%. Surprisingly, IL-U-HD not only improved the extraction efficiency but also enhanced the CEO cell viability and antioxidant performance [20].

##### Microwave-Assisted Extraction

Microwave-assisted extraction is a physical method that has received increasing attention due to its convenience, environmental friendliness, time-saving property, and high yield, and it has been widely used in the extraction of natural products. Jing et al. used the microwave-assisted extraction method to extract dietary polysaccharides from *Polygonatum sibiricum*. The microwave-assisted extraction process for *Polygonatum sibiricum* polysaccharides was developed as follows: a solid–liquid ratio of 1:30, a microwave radiation power of 700 W, and a microwave radiation time of 20 min. Under these conditions, the extraction rate of dietary polysaccharides from *Polygonatum sibiricum* was 6.71 ± 1.49% [21].

##### Freeze–Thaw Extraction Method

The freeze–thaw method is a technique that uses repeated freezing and thawing to form ice crystals in cells, and an increase in the salt concentration in the remaining liquid can cause cell rupture. The freeze–thaw method can maintain the integrity of the polysaccharide structure and has a high extraction rate, making it suitable for large-scale green industrial production. Wang et al. obtained the optimal extraction conditions for polysaccharides from *Polygonatum cyrtonema* Hua using the freeze–thaw method and response surface methodology, for which a liquid–solid ratio of 36.95:1, a freezing time of 4.8 h, and a thawing temperature of 55.99 °C were used. Under the optimal extraction conditions, the extraction rate of polysaccharides from *Polygonatum cyrtonema* Hua is 65.76 ± 0.32%. This extraction method is simple to operate and environmentally friendly, but it should be noted that it should not be used for proteins that are sensitive to temperature changes [22].

#### 2.1.2. Chemical Extraction of Polysaccharides from *Polygonatum sibiricum*

##### Hot Water Extraction

The hot water extraction method is a classic polysaccharide extraction method with the advantages of simple operation, convenient operation, a higher recovery rate of polysaccharides, better penetration, and harmlessness. In the process of hot water extraction, the yield of crude polysaccharides depends on the solid–liquid ratio, extraction temperature, span, extraction time, and other parameters. Using the hot water extraction method, polysaccharides isextracted from *Polygonatum kingianum* residue. The process conditions were as follows: an extraction temperature of 80 °C, an extraction time of 1.5 h, a solid–liquid ratio of 1:10 (*w*/*v*), and extraction twice Under these conditions, the yield of dietary polysaccharides from *Polygonatum kingianum* was 4.42% [23]. Hot water extraction is the most traditional extraction method but it has certain drawbacks, such as high energy consumption and long time consumption. However, due to its low cost and easy implementation, the hot water extraction method is more suitable for large-scale production.

##### Natural Deep Eutectic Solvent Extraction Method

Natural deep eutectic solvents (NADESs) have great potential as effective solvents for extracting polar and non-polar compounds and are non-toxic and green, have good biodegradability, and involve a simple preparation method. Zhang et al. optimized the extraction conditions of dietary polysaccharides from *Polygonatum sibiricum* using the response surface methodology as follows: NADESs were synthesized at a 1:2 choline chloride glycerol molar ratio and then extracted for 60 min at 60 °C with a liquid–solid ratio of 16.6 mL/g and a water content of 31.2%. The extraction rate of *Polygonatum sibiricum* dietary polysaccharide was 29.69%, which is 2.5 times that of traditional water extraction [24]. As a cheap, green, and free combination and new environmental solvent, deep eutectic solvents provide a new idea for extracting important, effective components in natural medicine.

#### 2.1.3. Enzyme-Assisted Extraction

Compared with hot water extraction, enzyme-assisted extraction releases polysaccharides and other substances by hydrolyzing cellulose and pectin in plant walls. Jing et al. used the enzymatic method to extract dietary polysaccharides from *Polygonatum sibiricum*. The experimental results show that under the conditions of an enzymatic hydrolysis pH of about 5.5, an enzymatic hydrolysis time of 2 h, an enzymatic hydrolysis temperature of 40 °C, an enzyme addition of 2%, and a solid–liquid ratio of 1:30, the yield of the enzyme-assisted polysaccharide extraction was 12.00 ± 3.26% [21]. The results show that compared with traditional extraction methods, the extraction rate of polysaccharides is greatly improved.

Overall, the hot water extraction method is still more suitable for large-scale industrial production. Compared to other methods, the hot water extraction method is easy to implement, has a low cost, and is relatively efficient at extraction. Nevertheless, it often contains protein after extraction, and further protein removal operations are needed. Ultrasonic-assisted extraction and enzyme-assisted extraction are more efficient and suitable compared to hot water extraction methods. Among the recent new methods for extracting polysaccharides from *Polygonatum sibiricum*, natural deep eutectic solvents and microbial extraction are more advanced. Researchers can choose appropriate methods to extract dietary polysaccharides from *Polygonatum sibiricum* based on the extraction purpose and the advantages and disadvantages of the extraction method. However, most importantly, different methods need to be developed in food industry production based on the specific types of polysaccharides required in order to achieve efficient extraction while minimizing waste disposal. The effects of different extraction methods on the extraction efficiency of *Polygonatum sibiricum* polysaccharides are shown in Table 1.

### 2.2. Study on Separation Technology of Dietary Polysaccharides from Polygonatum sibiricum

Under most conditions, the combination of the anion exchange column and gel column is the most commonly used separation method of dietary polysaccharides in order to obtain polygonatum polysaccharides through more refined separation. DEAE-52 cellulose and DEAE agarose gel rapid-flow chromatography columns are the two main anion exchange columns used to separate neutral and acidic polysaccharide components [28].

In order to purify polysaccharides, Cheng et al. used the DEAE agarose column to separate three crude polysaccharides, obtaining one neutral polysaccharide and one acidic polysaccharide. From the results, it can be seen that the crude polysaccharides of *Polygonatum cyrtonema* are mainly neutral polysaccharides, accounting for 68.48% [29]. Currently, besides the two efficient separation techniques mentioned above, the most commonly used methods for separating dietary polysaccharides from *Polygonatum sibiricum* are the ethanol precipitation method and the macroporous resin method. Bu et al. used anhydrous ethanol to separate crude polysaccharides. During the research process, anhydrous ethanol was added to the extract in a ratio of 1:4 and precipitated for 12 h. The precipitate in a water solvent was dissolved and deproteinized using a chloroform/n-butanol mixture (4:1, *v*/*v*) at a polysaccharide-to-organic-solvent ratio of 1:4 (*v*/*v*) [23]. Compared with chromatography, the graded precipitation method appears to be more convenient, but it takes more time and still needs to be optimized and explored in order to achieve industrial production as soon as possible.

### 2.3. Study on Purification Technology of Polygonatum sibiricum Dietary Polysaccharides

For the extraction of *Polygonatum sibiricum* polysaccharides, the hot water extraction method is usually used, but the crude polysaccharides obtained usually contain a large amount of protein. Excessive protein contents can affect the activity of dietary polysaccharides in *Polygonatum sibiricum*, so it is necessary to deprotein and further remove impurities to improve the purity of dietary polysaccharides in *Polygonatum sibiricum*. In traditional deproteinization, the ethanol precipitation, macroporous resin adsorption, Sevage, TCA, hydrochloric acid, and salt-out methods are mostly used, but most of these methods are inefficient and time-consuming, and most of them involve harmful organic solvents.

Currently, researchers are using ionic liquid tetrabutylammonium bromide (TBABr) to prepare an ionic liquid aqueous two-phase system (ILATPS) for separating polysaccharides and proteins extracted from roots and stems. The experimental results show that the extraction efficiencies of protein and polysaccharides in ILATPS are 98.6% and 93.5% [30]. This method is environmentally friendly and easy to operate, and it provides a reference for the study of modern *Polygonatum sibiricum* dietary polysaccharide deproteinization and also provides a practical strategy for the purification of plant-derived polysaccharides.

Surprisingly, the polysaccharides extracted from *Polygonatum sibiricum* were deproteinized using enzymatic methods, exhibiting strong specificity, a high deproteinization efficiency, and minimal polysaccharide loss. In the study on the deproteinization and antioxidant properties of dietary polysaccharides from *Polygonatum sibiricum*, Wang et al. used a polyethylene glycol ammonium sulfate dihydrate system to separate dietary polysaccharides from proteins, allowing the proteins to be enriched in the polyethylene glycol phase and the polysaccharides to be based on the ammonium sulfate phase. Wang et al. optimized the process using the response surface methodology and achieved a polysaccharide recovery rate of 91.29% under optimal conditions [31].

Based on the above results, from the scale point of view, most of methods are only suitable for small-scale processing in the laboratory and cannot meet the requirements of large-scale production. From the efficiency point of view, the preparation of an ionic liquid aqueous two-phase system by using ionic liquid tetrabutylammonium bromide obviously has greater potential, but due to its complexity, extensive experiments and research are still needed to lay the foundation for subsequent large-scale production. Undoubtedly, there is an urgent need for safe and harmless methods of purifying polysaccharides in green and environmentally friendly food industry production to meet consumers’ demand for *Polygonatum sibiricum* polysaccharide food.

## 3. Structural Characterization of *Polygonatum sibiricum* Dietary Polysaccharides

*Polygonatum sibiricum* dietary polysaccharides are a kind of macromolecular polymer, which has been studied for its special physical and chemical properties and biological activities. Its structural characteristics mainly include molecular-weight distribution, the types and proportions of monosaccharides, and the location of glucoside bonds. Table 2 summarizes the molecular weight, monosaccharide composition, and sugar chain structure of GPP.

### 3.1. Determination of Molecular Weight

The biological activity and structural properties of polysaccharides are closely related to their molecular weight, and determining the molecular weight is the basis for accurately studying the correlation between the polysaccharide structure and activity. Studies have demonstrated that the molecular-weight range of *Polygonatum sibiricum* dietary polysaccharides is mainly between 8.82 × 10^3^ and 6.3 × 10^7^ Da. This difference in molecular weight is mainly attributed to the source materials, the amount of mixture, the separation equipment, and other factors. Currently, high-performance gel permeation chromatography (HPGPC) has the advantages of a high resolution and high separation speed and is widely used to determine the molecular-weight distribution.

Interestingly, Wu et al. used HPGPC to detect the molecular-weight distribution of *Polygonatum sibiricum* dietary polysaccharides via the steaming process. The results show that the *Polygonatum sibiricum* dietary polysaccharides had two peaks at 0 h, with molecular weights of 3.348 × 10^5^ Da and 7.074 × 10^3^ Da, in the first four hours of cooking, and both peaks increased at different degrees. Especially, the longer cooking time led to the decrease in the molecular weight of the *Polygonatum sibiricum* dietary polysaccharides. Compared with the dietary polysaccharides of *Polygonatum sibiricum* cooked for 4 h, the molecular weight of peak 1 in the *Polygonatum sibiricum* dietary polysaccharides significantly decreased by 86.8% after 6 h of cooking. Surprisingly, when the cooking time exceeded 6 h, peak 2 disappeared. The results show that cooking for 2 h is beneficial to keep the polysaccharide content in the rhizome, which is better than cooking for 4 h. However, a long cooking time (≥6 h) not only leads to the destruction of PCPs but also reduces their immunomodulatory activity [38].

### 3.2. Analysis of Constituent Monosaccharides

Understanding the monosaccharide composition is crucial for analyzing the structural characteristics and product quality control of *Polygonatum sibiricum* polysaccharides. It reveals the relative composition of dietary polysaccharides in *Polygonatum sibiricum* and further confirms the structure of the polysaccharides. The process of detecting the monosaccharide composition usually involves hydrolyzing polysaccharides into monosaccharides and then detecting the released monosaccharides or their derivatives. PMP derivatization is the most widely used derivatization reagent in monosaccharide detection, but there are still some drawbacks, especially that it is not suitable for measuring ketoses like fructose (Fru).

Therefore, non-derivatization detection methods are often used to supplement the analysis of monosaccharides composed of *Polygonatum sibiricum* dietary polysaccharides. The proportion of monosaccharides in different polysaccharides of the Polygonatum genus varies, and they mainly include rhamnose (Rha), arabinose (Ara), glucose (Glc), ribose (Fuc), ribose (Rib), galactose (Gal), xylose (Xyl), mannose (Man), galacturonic acid (GalA), and glucuronic acid (GlcA) [16]. Zhao et al. used the PMP-HPLC and HPLC-RID methods to determine the mannose (Man), glucuronic acid (GlcA), rhamnose (Rha), and galacturonic acid (GalA) of *Polygonatum sibiricum, Polygonatum kingianum, Polygonatum cyrtonema,* and *Polygonatum odoratum* for the further analysis and detection of the reactants. The results show that *Polygonatum sibiricum*, *Polygonatum cyrtonema*, and *Polygonatum kingianum* polysaccharides are mainly composed of fructose, galacturonic acid, and galactose and contain a small amount of rhamnose, arabinose, xylose, and glucose. Interestingly, the polysaccharides of *Polygonatum odoratum* are mainly composed of fructose and trace galacturonic acid, galactose, rhamnose, arabinose, xylose, and glucose [40]. These data indicate that *Polygonatum sibiricum* polysaccharides are heteropolysaccharides mainly composed of fructose and have great potential in the food industry.

### 3.3. Glycoside Bonds of Polygonatum sibiricum Dietary Polysaccharides

Research has found that different types and components can lead to different glycosidic bonds in the dietary polysaccharides of *Polygonatum sibiricum*. Not only the molecular weight and monosaccharide composition but also the glycosidic bonds of polysaccharides have a close association with their biological activity. Common techniques for identifying glycosidic bonds include partial acid hydrolysis, electrospray ionization (ESI) mass spectrometry, enzyme digestion, methylated gas chromatography–mass spectrometry, and nuclear magnetic resonance spectroscopy. Currently, high-throughput solid-phase methylation analysis is also considered a powerful technique for the qualitative analysis of natural carbohydrates.

FT-IR shows that the main absorption peaks of these polysaccharides are very similar. The vibration absorption peak of O-H is approximately 3402 cm^−1^. Surprisingly, the C-H stretching of CH, methylene, and the methyl groups is 2939 cm^−1^ and 2891 cm^−1^, with an absorption peak of approximately 1740 cm^−1^, located in the C=O stretching vibration of the esterified carboxyl group (COO-R). Due to the asymmetric stretching and asymmetric stretching (COO-) of carboxylic acid anions, absorption peaks appear around 1640 cm^−1^ and 1420 cm^−1^. It is interesting that they differ due to their varying degrees of esterification. The ratio of the peak area at 1740 cm^−1^ (corresponding to the esterified carboxyl group) to the sum of the peak areas at 1740 and 1640 cm^−1^ (corresponding to the total carboxyl group) can be approximately calculated as the degree of esterification. Among them, the degree of esterification of PM is the lowest. In addition, the PA and PCI are higher than other polygonatum polysaccharides, and the weak peak is located at 766 cm^−1^, which is caused by the skeleton bending of the galactose ring [16]. Surprisingly, the absorption peaks at 1638.44, 1417.04, 1129.94, and 1029.16 cm^−1^ were attributed to C=O, COOH, C-OH, and C-O-C stretching vibrations, indicating the presence of uronic acid and furan sugar rings. The absorption peak at 878.19 cm^−1^ is considered to be an alpha glycosidic bond. In addition, the peaks at 933.09 and 818.12 cm^−1^ are attributed to the furan sugar ring with β-glycosidic bonds. NMR results show that the →2)-β-D-Fruf-(1→2)-β-D-Fruf-(1→link existed in *Polygonatum sibiricum* polysaccharides [41].

Currently, there are some difficulties in the structural characterization of plant polysaccharides, and a single analytical technique has limitations. Surprisingly, methylation chemical analysis can determine monosaccharide linking sites, but it cannot resolve glycosidic bond configurations (α/β type) and polysaccharide sequence information. However, periodate oxidation can be used to infer the sugar chain linking types but has limited ability to resolve branching structures or acid-resistant bonds (such as furan glycosides). Although some acid hydrolyses can generate shortchain fragments, the selection of the cleavage sites depends on experience and may miss key structural information. Moreover, nuclear magnetic resonance (NMR) and X-ray diffraction require highly purified and structurally homogeneous polysaccharide samples, but natural polysaccharides often have microscopic heterogeneity (such as molecular-weight dispersion and sugar chain defects), which leads to vague results of advanced structure analysis. High-performance gel permeation chromatography (HPGPC) is often used for purity assessment, but it is impossible to distinguish polysaccharide mixtures with similar molecular weights (such as the coexistence of different sugar chain branches), which may be misjudged as a single component [42]. The research suggests a solution for multitechnology joint analysis, such as the precise analysis of shortchain fragments generated by partial acid hydrolysis through NMR and mass spectrometry, and the reduction in the overall linkage patterns through serial methylation analysis. Simultaneously, enzymatic hydrolysis chromatography was used to selectively cleave key positions through specific glycosidases, combined with HPGPC or MALDI-TOF MS to track the degradation products, which can verify the branching structure and sequence [43]. The further integration of multidimensional data and computational model assistance techniques can be used to construct computational models by integrating multiple sources of data (such as methylation, NMR, enzymatic hydrolysis products) to infer structural features that have not been directly captured by experiments (such as dynamic conformational changes). Overall, a single characterization technique is limited by the method principles and sample complexity, which can easily lead to structural misjudgment or information loss. Only through the systematic combination of chemical modification, physical spectroscopy, biological enzymatic hydrolysis, and computational models can the comprehensive analysis of polysaccharide structures be achieved, promoting their application in drug development and functional materials.

## 4. Pharmacological Activity of *Polygonatum sibiricum* Dietary Polysaccharides

### 4.1. Hypoglycemic Activity

**Figure 3:**
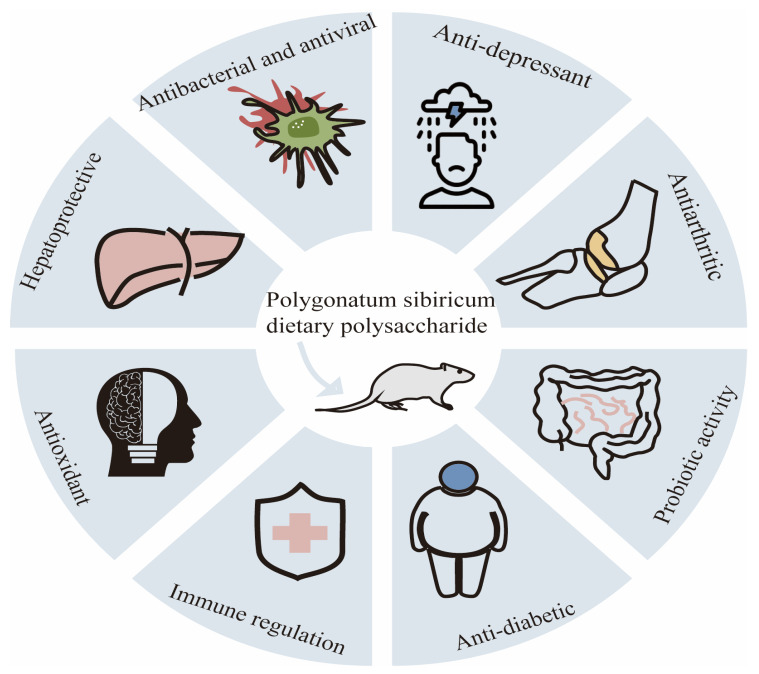
Multiple physiological activities of *Polygonatum sibiricum* polysaccharides.

According to the data of the International Diabetes Federation, the global prevalence of diabetes among adults aged 20–70 years is estimated to be 10.5%, affecting 536.6 million people. It is expected that by 2045, this number will rise to 12.2%, and approximately 783.2 million people will be affected [44]. Diabetes mellitus (DM) is a metabolic disease characterized by lipid and carbohydrate metabolism disorder. The disease is mainly divided into type 1, type 2, and pregnancy-type diabetes. The etiology of diabetes is mainly attributed to the combination of genetic and environmental factors, mainly due to insulin deficiency or insufficient insulin secretion, leading to glucose, lipid, and protein metabolism disorder [45]. Surprisingly, the current research has shown that dietary polysaccharides from *Polygonatum sibiricum* have hypoglycemic activity. In diabetes rats, polygonatum polysaccharides can improve clinical symptoms (restlessness, overeating, polyuria, and weight loss), increase the plasma insulin and C-peptide levels [46], and slow down the progression of diabetes retinopathy and cataracts by reducing the fasting blood glucose and glycosylated hemoglobin levels and inhibiting the oxidative stress reaction. Researchers have found that dietary polysaccharides from *Polygonatum sibiricum* can reduce the insulin resistance index, increase the OGTT and serum insulin levels, lower the free fatty acid content to improve lipid metabolism, and reduce the glycosylated serum protein content to enhance glucose metabolism in T2DM mice, leading to a decrease in blood sugar [46]. Xie et al. extracted a homogeneous polysaccharide (PSPW) from *Polygonatum sibiricum*, which can improve the glucose tolerance, insulin sensitivity, and liver glucose metabolism of type 2 diabetes mice. Compared with the model group, PSPW can increase the phosphorylation levels of PI3K and AKt, and it can effectively inhibit the increase in the phosphorylation levels of FoxO1 and GSK3 β in T_2_D mice. These results suggest that PSPW may reduce liver glucose production by activating the PI3K/Akt signaling pathway [47]. The hypoglycemic effect of *Polygonatum sibiricum* dietary polysaccharides enables them to be used as main drugs or auxiliary materials in the pharmaceutical and health food industries. The various physiological activities of *Polygonatum sibiricum* polysaccharides are shown in Figure 3.

### 4.2. Antioxidant Activity

In biological systems, oxidative stress refers to a state of imbalance between the oxidative and antioxidant effects in the body, which is a negative effect produced by free radicals in the body and may lead to various diseases such as cardiovascular disease and neurodegenerative diseases. Zheng et al. found that *Polygonatum sibiricum* dietary polysaccharides can significantly improve the learning and memory abilities of aging mice and reverse the pathological changes in the renal tissue by reducing oxidative stress and balancing calcium and phosphorus metabolism [48]. Surprisingly, the research results of Li et al. show that the free radical scavenging activity of dietary polysaccharides from *Polygonatum sibiricum* significantly increased after steaming and gradually increased with the increase in the steaming times [49]. Moreover, Zhao et al. used two models for scavenging DPPH radicals and hydroxyl radicals to detect the free radical scavenging rate of *Polygonatum cyrtonema* dietary polysaccharides processed in different ways [50].

### 4.3. Immunomodulatory Activity

With the change in the living environment, people have begun to pursue physical conditions that can cope with harsh environments. The immune system is the body’s main defense system, which helps to destroy abnormal cells (such as cancer cells) and prevent pathogens and foreign molecules. The dietary polysaccharides of *Polygonatum sibiricum* have rich biological activity, especially immune regulatory ability, which meets the requirements of safety, harmlessness, and significant functionality for people. *Polygonatum sibiricum* has a regulatory effect on the immune system, mainly through activating the immune system, improving the growth and activity of immune cells, and promoting antibody synthesis and other pathways to exert immune enhancement effects. Currently, studies have shown that dietary polysaccharides from *Polygonatum sibiricum* can exert their immune regulatory pathways through various signaling pathways and can act on multiple targets both in vivo and in vitro [51]. NF-κB is a key immune signaling pathway, known as the central regulatory factor of the immune response, which can promote the expression of various molecules involved in the immune process. Interestingly, the immunomodulatory effect of dietary polysaccharides from *Polygonatum sibiricum* on macrophages is related to the activation of the NF-κB signaling pathway [52]. Similarly, polysaccharides extracted from *Polygonatum sibiricum* can activate macrophages in vivo and enhance their function through the NF-κB pathway [53].

### 4.4. Anti-Osteoporosis Activity

Osteoporosis is a common degenerative joint disease in the world and one of the diseases currently faced by many middle-aged and elderly people. Its characteristics include pain, swelling, stiffness, and impaired joint movement in areas such as the knee and hip joints. Compared to people without osteoarthritis, the prevalence of osteoarthritis slightly increases the risk of cardiovascular death in patients. More than half of elderly arthritis patients suffer from other chronic diseases, indicating that the pain of osteoarthritis will worsen accordingly [54]. *Polygonatum sibiricum* dietary polysaccharides have shown inhibitory effects on IL-1 β-induced chondrocyte inflammation and the TLR2/NF-κB signaling pathway in in vitro experiments and have the ability to regulate cartilage matrix metabolism. In in vivo experiments, dietary polysaccharides from *Polygonatum sibiricum* inhibited the activation of the TLR2/NF-κB signaling pathway in mouse knee cartilage induced by medial meniscus instability and reduced the levels of serum inflammatory cytokines. Overall, dietary polysaccharides from *Polygonatum sibiricum* can be used as a new type of green, natural, environmentally friendly, and low-toxicity drug for the treatment and prevention of osteoarthritis [10].

### 4.5. Antidepressant Activity

Depression is a common neurological and psychiatric disorder with a high incidence and high clinical cure rate but a low treatment acceptance rate and high recurrence rate which is characterized by a persistent low mood and sometimes suicidal thoughts or behaviors. Current cell and animal experiments have shown that some polysaccharides have antidepressant effects [55]. In recent research, lipopolysaccharides and chronic unpredictable mild stress were used to establish a depressed mouse model, detect the antidepressant effect of *Polygonatum sibiricum* dietary polysaccharides (PSPs), induce depressive-like behavior by injecting lipopolysaccharide (LPS), reduce the hippocampal 5-hydroxytryptamine (5-HT) levels, increase the serum cortisol (CORT) levels and hippocampal oxidative stress, and promote the activation and inflammatory response of ERK1/2, NF-κB, and GFAP. Surprisingly, the administration of *Polygonatum sibiricum* dietary polysaccharides reduces these changes and prevents depression-like behavior. The experimental results suggest that *Polygonatum sibiricum* dietary polysaccharides may prevent depressive-like behaviors as well as synaptic and neuronal damage by reducing ROS/HPA axis hyperfunctioning and inflammatory responses [56]. Interestingly, Shen et al. found in their study that *Polygonatum sibiricum* dietary polysaccharides can reduce depressive behavior in mice by inhibiting oxidative stress, and that *Polygonatum sibiricum* dietary polysaccharides can prevent chronic unpredictable mild stress and reduce depressive behavior by inhibiting the calpain system, nuclear erythropoietin 2 related factor 2, and NOD like receptor protein 3 (NLRP3). Therefore, the results indicate that *Polygonatum sibiricum* dietary polysaccharides may exert antidepressant effects by regulating the stress calpain 1-NLRP3 signaling axis [57]. Currently, most depression treatment drugs are chemical drugs that can cause varying degrees of damage to the human body within a certain range. As a traditional Chinese medicine extract, *Polygonatum sibiricum* dietary polysaccharides not only have a therapeutic effect on depression but also do not cause damage to the body or environment.

### 4.6. Liver-Protecting Activity

The liver plays a central role in the body’s physiological processes, participating in the regulation of carbohydrate, lipid, and protein metabolism to maintain the energy balance and physiological functions. In addition, the liver is crucial in its function of synthesis, including the production of proteins, glycogen, and bile, ensuring the balance of the plasma components. Currently, some studies have demonstrated the hepatoprotective effect of dietary polysaccharides extracted from *Polygonatum sibiricum* roots and stems and have modeled experiments using CCL4. CCL4 induces experimental liver injury by activating the production of free radicals and reactive substances mediated by cytochrome P-450.

Homogeneous components of *Polygonatum sibiricum* dietary polysaccharides showed different protective effects on the cell viability and biochemical indexes of supernatant (10–100 μg/mL), which indicated that they had potential protective effects on alleviating liver injury. This effect is dose-dependent, and the higher the dosage of dietary polysaccharides in *Polygonatum sibiricum* (100 μg/mL), the better the protective effect. Different treatment groups have different effects on various indicators in different concentration ranges, indicating that different components may have different degrees of liver protection [58]. Liu et al. found that Lactobacillus cannot enhance high-fat- and high-sucrose-induced liver injury, but the metabolites produced by Lactobacillus cultured with dietary polysaccharides from *Polygonatum sibiricum* can protect against high-fat- and high-sucrose-induced liver injury, impaired liver lipid metabolism, and the dysbiosis of the gut microbiota. Importantly, this provides a new technology for the combination of Lactobacillus rhamnosus and dietary polysaccharides from *Polygonatum sibiricum* for the prevention of unhealthy diet-induced metabolic disorders and liver damage diseases, and for the development of probiotic products [59].

**Figure 4:**
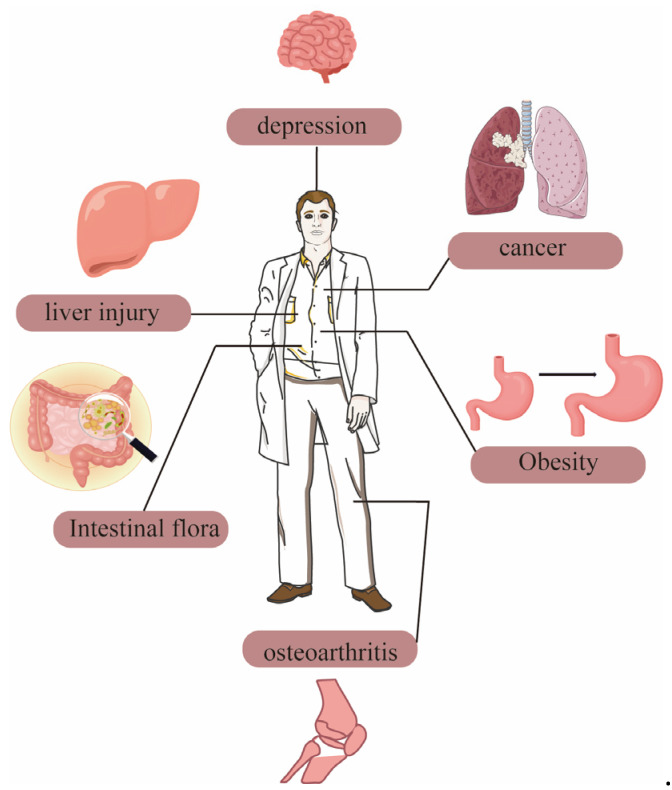
The functional application of *Polygonatum sibiricum* dietary polysaccharides in humans.

The protective effect of dietary polysaccharides from *Polygonatum sibiricum* on the liver make them a natural product with great potential in the future health food industry. The functional application of *Polygonatum sibiricum* dietary polysaccharides in humans is shown in Figure 4.

### 4.7. Other Activities

In previous studies, *Polygonatum sibiricum* dietary polysaccharides have also been found to have other activities, such as probiotic, antibacterial [60], antiviral, and anti-tumor activities. Luo et al. determined the changes in the short-chain fatty acids in mouse feces by GC-MS during the study of *Polygonatum sibiricum* dietary polysaccharides, and they further verified the correlation between short-chain fatty acids and intestinal flora. The experimental results show that *Polygonatum sibiricum* dietary polysaccharides can regulate intestinal flora by increasing short-chain fatty acids [13].

Hu et al. show that dietary polysaccharides from *Polygonatum sibiricum* can significantly regulate the richness and diversity of microbial communities during in vitro digestion and fermentation, especially Clostridium perfringens and Bacteroidetes, both of which can convert carbohydrates into short-chain fatty acids, providing multiple benefits for regulating human metabolism and intestinal health [61].

In most cases, researchers have linked tumors with inflammation. Although uncontrollable inflammation is not the initial cause of complex diseases such as tumors, many tumors are induced at the site of inflammation, and there is a close relationship between inflammation and some tumors. Toll-like receptors (TLRs) can selectively recognize pathogen-related conserved structures in pathogens and pre-activate natural immunity, which are important activators of the host innate immunity. TLR4 plays a core role in combating bacterial infections. The experimental results of Long et al. show that after treatment with *Polygonatum sibiricum* dietary polysaccharides on female mice, the tumor weight significantly decreased, the tumor inhibition rate increased, and the organ index increased. Interestingly, the proportion of lymphocytes and the level of serum cytokines is significantly improved. *Polygonatum sibiricum* dietary polysaccharides play an immune enhancement role against lung cancer by activating the TLR4 receptor and downstream MAPK/NF-κ B signaling pathway [52].

Currently, the research on the pharmacological activities of *Polygonatum sibiricum* dietary polysaccharides is still deepening, and all pharmacological activities of *Polygonatum sibiricum* dietary polysaccharides have developed to varying degrees. Among them, hypoglycemic and immunomodulatory activities are mostly studied, but their underlying mechanisms are still unknown. Therefore, more animal studies and clinical trials should be conducted in order to better explain the mechanisms and effects and promote the development of *Polygonatum sibiricum* dietary polysaccharides in food, cosmetics, and other fields. The specific concentration range or IC50 of *Polygonatum sibiricum* dietary polysaccharides for pharmacological activity is shown in Table 3.

## 5. Application Status of *Polygonatum sibiricum* Dietary Polysaccharides in Food and Other Fields

**Figure 5:**
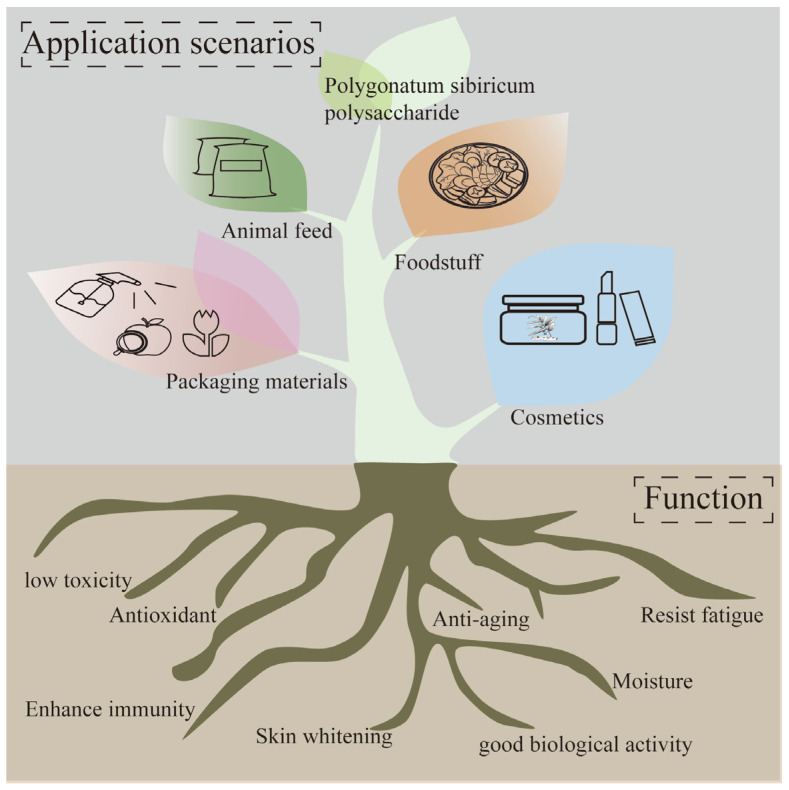
Application of *Polygonatum sibiricum* polysaccharides.

*Polygonatum sibiricum* dietary polysaccharides, as a natural plant-derived substance, have the characteristics of good biological activity, low toxicity, and cleanliness and have great application potential in the food, pharmacy, cosmetics, and feed fields. These characteristics enable *Polygonatum sibiricum* dietary polysaccharides to be widely used in the food chain, as packaging in food, or directly as functional ingredients such as flavoring agents or antioxidants. The application of *Polygonatum sibiricum* polysaccharides is shown in Figure 5.

### 5.1. Food Field

After the Chinese Ministry of Health released the list of drugs and the food homology in 2002, many traditional Chinese medicines, including *Polygonatum sibiricum*, are included in food products to enhance or improve their functional properties. *Polygonatum sibiricum* and its extracts are widely used as raw materials in various food formulas to make various products, mainly including wine, beverages, biscuits, nutritional soups, fructose wrappers, and dairy products. With the increasing demand for functional foods from consumers, there has been a surge in *Polygonatum sibiricum*-related products on the market. However, due to the inaccurate processing and insufficient utilization of *Polygonatum sibiricum*, *Polygonatum sibiricum* dietary polysaccharides have not found suitable application areas for themselves. In terms of the extraction methods of dietary polysaccharides from *Polygonatum sibiricum*, different processing and extraction methods may alter its main pharmacological activity, and the simple extraction methods used in the food industry may hinder the application of dietary polysaccharides from *Polygonatum sibiricum* [21]. Specifically, when the food industry applies extraction methods with higher technological contents and stricter requirements, the cost of producing food may correspondingly increase, which can reduce or shrink the market demand among the target audience. Research has shown that dietary polysaccharides from *Polygonatum sibiricum* can enhance texture and retain moisture in food, which is different from the foaming or emulsifying properties of *Polygonatum sibiricum* saponins. Therefore, in the food industry, it is necessary to reasonably utilize the characteristics and functions of *Polygonatum sibiricum* dietary polysaccharides to develop green, natural, and harmless functional foods [62].

#### 5.1.1. Drinks

Carbohydrates, as additives in various beverages, mainly play a seasoning role. But a plethora of sugar increases the risk of diabetes. However, in recent years, there has been an upward trend in people’s demand for sugar-free or low-sugar products, which is particularly prominent among individuals who engage in fitness activities and those suffering from diabetes. *Polygonatum sibiricum* dietary polysaccharides, as natural high-molecular-weight extracts, can not only regulate odors but also improve human functions without causing side effects in various groups. Especially, the *Polygonatum sibiricum* raspberry health drink has antioxidant activity. The *Polygonatum sibiricum* instant tea prepared by Wang et al. also has in vitro antioxidant activity, and its ability to scavenge free radicals increases with its concentration [63]. When *Polygonatum sibiricum* dietary polysaccharides are used as additives in beverages, they not only have antioxidant effects but also have certain immune-enhancing effects in vitro and in vivo [64]. These results indicate that dietary polysaccharides from *Polygonatum sibiricum* can serve as a good health beverage additive and have a large audience and application market.

The research results of Song et al. indicate that the secondary metabolites in “Huangjin” beverages are mainly polyphenolic substances, including phenolic acids, flavonoids, and alkaloids. Compared with “Huangjin” tea, “Huangjin” wine contains more types of flavonoids and a higher flavonoid content. Homoisoflavones are considered to be uncommon flavonoids in nature, and they have been reported to show a wide range of biological activities, including antimutagenic, immunomodulatory, anti-diabetes, etc., activities, indicating that polygonatum polysaccharide beverages have health-promoting effects [65].

In addition to antioxidant and immune-regulating effects, *Polygonatum sibiricum* polysaccharides can also reduce the occurrence of chronic diseases such as diabetes and arthritis. As a natural green food ingredient, the combination of *Polygonatum sibiricum* dietary polysaccharides with other foods enhances the nutritional value and health benefits of the foods. Surprisingly, *Polygonatum sibiricum* dietary polysaccharides can be processed into different products based on their multifunctional properties to cater to consumers’ different preferences.

#### 5.1.2. Functional Food

Functional food mainly has the function of regulating the body, will not cause any acute, subacute, or chronic harm to the human body and is mainly used for the purpose of supplementing vitamins, minerals, etc. At present, the polysaccharide health foods sold on the market mainly include dietary fiber, shiitake mushroom polysaccharides, etc., while *Polygonatum sibiricum* polysaccharides are often mixed with other drugs to prepare tablets, pastes, tablet candies, and other forms for sale. *Polygonatum sibiricum* polysaccharide products often have health functions such as hypoglycemic, immunity-enhancing, and anti-fatigue effects.

Xian et al. studied the effect of dietary polysaccharides from *Polygonatum sibiricum* on alleviating fatigue caused by excessive exercise and demonstrated through mouse swimming fatigue experiments that increasing the swimming time leads to elevated levels of creatine kinase and malondialdehyde, as well as decreased levels of lactate and blood urea nitroge [66]. The Jiuzhuanhuangjing Pill prepared by Shen et al. prevents mitochondrial structural damage and functional disorders by reducing oxidative stress, thereby preventing cell death and enhancing energy metabolism, improving fatigue in both in vivo and in vitro models, preventing cell death, and enhancing energy metabolism [67].

Chen et al. conducted an in vitro cecal fermentation experiment that showed that during the fermentation process, most of the pentachlorophenol in fecal samples from healthy and obese individuals is degraded by the gut microbiota. The utilization of pentachlorophenol by the gut microbiota leads to the production of a large amount of short-chain fatty acids (SCFAs), which lowers the environmental pH value, stimulates the proliferation of beneficial bacteria, and prevents the growth of harmful bacteria [68]. In summary, *Polygonatum sibiricum* polysaccharides have significant beneficial effects, such as anti-fatigue and gut microbiota-promoting effects. They can be used as an ideal source of functional food to reduce fatigue or improve the gut microbiota, effectively enhancing the nutritional value of food and promoting health.

### 5.2. Food Packaging

Currently, plastic packaging products have become increasingly common, leading to a large amount of plastic waste. If no measures are taken, it will result in more serious environmental pollution. In recent years, people have increasingly attached importance to food and environmental safety, and the application of edible coatings and packaging films in the food industry has begun to increase. As a new type of material, edible coatings aim to extend the shelf life of food and protect the environment from plastic damage via the addition of antioxidants, antibacterial agents, or other active compounds to packaging materials [69]. Surprisingly, *Polygonatum sibiricum* has received widespread attention due to its significant antioxidant and antibacterial properties, making it a research topic in food packaging.

**Figure 6:**
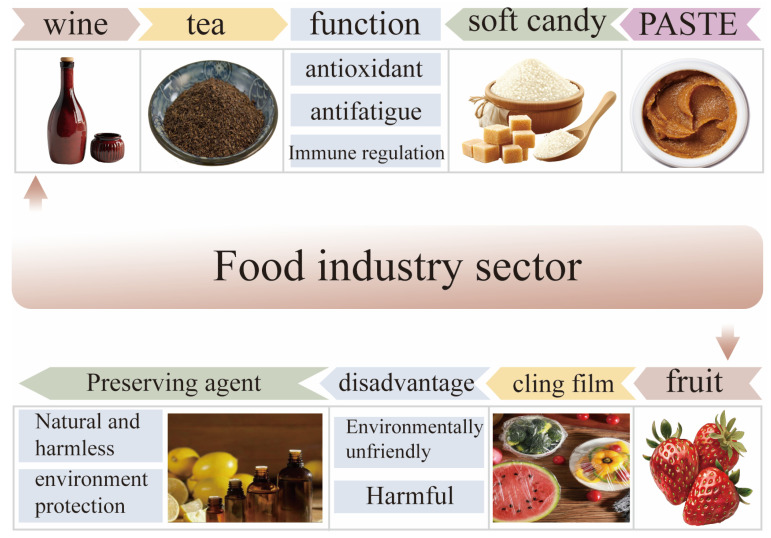
Application of *Polygonatum sibiricum* dietary polysaccharides in the food industry.

Li et al. developed a composite material using dietary polysaccharides from *Polygonatum sibiricum* containing konjac glucomannan and prepared a new type of active food packaging film. The results show that the addition of *Polygonatum sibiricum* extract could improve the crystallinity of the composite film. When the dietary polysaccharide content of *Polygonatum sibiricum* is 0.2%, the tensile strength of the composite film reaches its optimal level. Nevertheless, when the content of *Polygonatum sibiricum* dietary polysaccharides is less than 0.2%, it has good barrier properties, UV-shielding properties, and antibacterial properties. Interestingly, the composite material can reduce the weight loss of strawberries and extend their shelf life, improving their overall quality [70]. In summary, *Polygonatum sibiricum* dietary polysaccharides, as active packaging materials, have good preservation effects due to their antioxidant activity. The preservation effect of *Polygonatum sibiricum* dietary polysaccharide extract is related to its concentration. If the concentration is too high, it may increase the thickness of the film and hinder the exchange of gas and water between foods (such as fruits). In the development process of food packaging composite materials, it is necessary to explore suitable concentration ranges in order to better apply them in the field of food packaging materials. The application of *Polygonatum sibiricum* dietary polysaccharides in the food industry is shown in Figure 6.

### 5.3. Cosmetic Field

The demand for skincare products containing natural substances in the cosmetics industry has significantly increased, with over 75% of cosmetics currently containing natural plant extracts [71]. Many brands use cosmetics rich in natural ingredients as their main selling point [72].

Dietary polysaccharides from *Polygonatum sibiricum*, a plant known for its multiple functional characteristics, has great potential in the development of cosmetics, especially its whitening, wrinkle prevention, moisturizing, and anti-aging properties. Research has shown that the active substances in *Polygonatum sibiricum* have significant antioxidant activity, effectively eliminating intracellular free radicals and exerting anti-aging effects. Surprisingly, dietary polysaccharides from *Polygonatum sibiricum* have been shown to protect the skin, reduce the lipofuscin content, and significantly delay skin aging in mice [73]. Theoretically, polysaccharides containing a large number of hydrophilic groups can bind to water through hydrogen bonds, providing immediate moisturizing effects and forming a protective film on the skin surface, reducing water evaporation. In most cases, all the active substances with antioxidant and antibacterial effects in *Polygonatum sibiricum* dietary polysaccharides can be used as cosmetic additives. Therefore, *Polygonatum sibiricum* dietary polysaccharides have great application potential in the cosmetic industry.

### 5.4. Animal Feed Field

In traditional agriculture, some drugs may be used to maintain animal health, which may lead to drug residues in livestock and poultry products, posing a risk to human health. In recent years, as people have increasingly focused on the quality and safety of livestock and poultry products, their attention has shifted to natural products. Natural active substances in plants can become low-toxicity alternatives to some antibiotics or other drugs, providing another solution for them [74].

*Polygonatum sibiricum* dietary polysaccharides have a series of biological activities and are expected to be a potential feed additive. Shu et al. show that *Polygonatum sibiricum* dietary polysaccharides can effectively protect the structure and function of chicken immune organs, enhance the antioxidant and immune activities of chickens, and regulate the expression levels of related cytokines (IL-2, IL-6, IFN-γ), thereby preventing and improving chicken immune deficiency diseases; therefore, an appropriate amount of *Polygonatum sibiricum* dietary polysaccharide extract can enhance the immune and antioxidant functions of animals and enhance their resistance to harsh environments and pathogens [75]. Excessive addition may not achieve better results and can lead to waste; therefore, when exploring the application of *Polygonatum sibiricum* in feed, the content of *Polygonatum sibiricum* should be considered in order to better apply it in the field of animal feed.

## 6. Conclusions and Expectations

**Figure 7:**
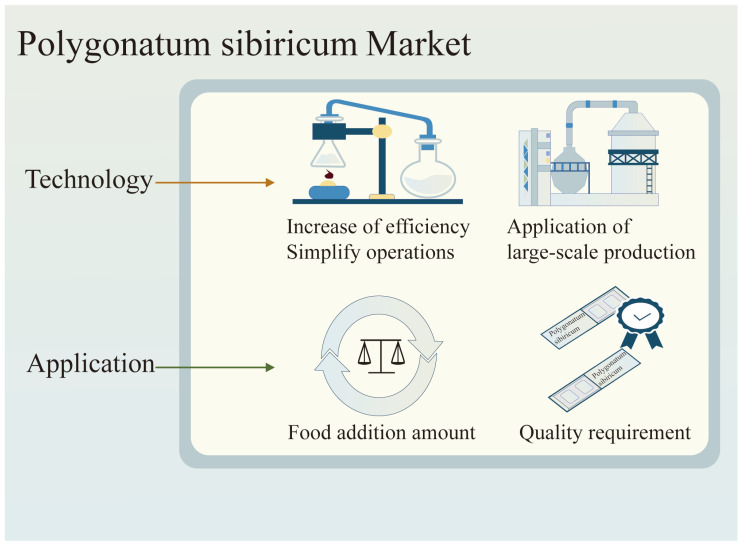
Problems that need improvement in *Polygonatum sibiricum* polysaccharides.

Dietary polysaccharides of *Polygonatum sibiricum* are the main active ingredients of *Polygonatum sibiricum* in the lily family. In recent years, it has attracted increasing attention due to its hypoglycemic and lipid-lowering effects. In addition, *Polygonatum sibiricum* also has pharmacological activities such as antioxidant, anti-aging, immune regulation, anti-osteoporosis, antidepression, liver protection, probiotic, antibacterial, antiviral, etc., properties, and *Polygonatum sibiricum* dietary polysaccharides have also been developed and utilized in different fields, such as *Polygonatum sibiricum* rice wine in the food and health fields and *Polygonatum sibiricum* oral liquid and Tangmaikang capsules in the pharmaceutical field. However, there are still some issues and many challenges that urgently need to be addressed. Firstly, there are some issues with the existing extraction techniques during the preparation process, such as low yields, low efficiency, cumbersome experimental methods, and inconvenient operation. In particular, we need to pay attention to environmental protection when preparing experimental materials in order to meet the world environmental protection theme. In the purification and separation process, in order to be used for large-scale production as soon as possible, the technical complexity and long-term consumption are still issues that need to be actively addressed, as shown in Figure 7.

Surprisingly, with the development of science and technology, people’s understanding of *Polygonatum sibiricum* polysaccharides is becoming increasingly clear, and the safety of food and drugs is gradually attracting widespread attention. *Polygonatum sibiricum*, as a medicinal and edible plant, has mainly been processed using the nine steaming and nine preparation methods in the history of traditional Chinese medicine. However, various new technologies may cause changes in the content and composition of *Polygonatum sibiricum* polysaccharides, so the processing and extraction techniques of *Polygonatum sibiricum* still need to be continuously explored. In addition, there are many literature studies on the biological activity and commercial application potential of dietary polysaccharides from *Polygonatum sibiricum*. However, their application as functional food ingredients has not been widely studied, and research on their biological activity when added to food is also limited. Moreover, it is necessary and urgent to control the use of dietary polysaccharides from *Polygonatum sibiricum* and the quality of related products. Therefore, the research on dietary polysaccharides in *Polygonatum sibiricum* needs to be widely studied.

## Figures and Tables

**Table 1:** Effects of different extraction methods on the extraction efficiency of *Polygonatum sibiricum* polysaccharides.

Source	Extraction Method	Molecular Weight (Da)	Extraction Efficiency	Characteristics	References
*Polygonatum sibiricum*	Ethanol ultrasonic extraction		5.55%	Potential to become AChE inhibitors for the treatment of Alzheimer’s disease	[17]
*Polygonatum sibiricum*	Enzyme-assisted extraction	3.926 × 10^3^	12.00%	Maintains lipid-lowering and antioxidant activities	[21]
*Polygonatum sibiricum*	Hot water extraction	4.025 × 10^3^	14.86	Maintains the antioxidant activity of polysaccharides	[21]
*Polygonatum cyrtonema*	Freeze–thaw method		65.76%	Significant antioxidant activity	[22]
*Polygonatum kingianum*	Natural deep eutectic solvent extraction method		29.69%	Maintains immune regulatory function	[24]
*Polygonatum sibiricum*	Ultrasound-assisted extraction Deep eutectic solvent	4.6 × 10^3^	43.61%	Maintains the antioxidant activity of polysaccharides	[25]
*Polygonatum sibiricum*	radiation-induced degradation and extraction		32.40%	Excellent reactive oxygen species scavenging ability	[26]
*Polygonatum sibiricum*	Water extraction Microwave-assisted degradation	2.33 × 10^3^	21.5%	Maintains good antioxidant activity	[27]

**Table 2:** Molecular weight, monosaccharide composition, and sugar chain of *Polygonatum sibiricum* dietary polysaccharides.

Category	Component Name	Molecular Weight (Da)	Monosaccharide Composition	Sugar Chain	References
*Polygonatum sibiricum*	PSP-N-b-1	6.3 × 10^3^	Man, GlcA, Rha, GalA, Glc and Ara		[32]
	PSP-N-b-2	5.78 × 10^3^			
	PSP-N-c-1	3.45 × 10^3^			
	PSP-NP	4.30 × 10^4^	Gal, Man, Glc	1,4-β-D-Gal, 1,4,6-β-D-Gal, T-α-D-Man, 1,4-α-D-Glc, T-α-D-Glc	[33]
	PSP-1	3.865 × 10^4^	Glc	→4-α-D-Glcp-1→backbone with the substitution at O-6 with the β-D-Glcp-1→residues	[34]
*Polygonatum kingianum*	PKP-s1	1.405 × 10^4^	Glc, Man, GalA, Gal, GlcA, Ara	polysaccharide component is mainly composed of glucose connected to (→1), (1→2), (1→6), and (1→4), as well as mannose connected to (1→2)	[35]
*Polygonatum cyrtonema*	NPCP	3.2 × 10^3^	Glu, Gal	→1)-α-D-Glc-(4→1)-β-D-Gal-(3→)	[36]
	DPC1	3.803 × 10^3^	Fru, Glc	(2→6)-linked β-D-Fruf residue backbone with an internal α-D-Glcp residue and two (2→1)linked β-D-Fruf residue branches	[37]
	PPC1	7.019 × 10^3^	Gal	(1→4)-β-D-galactan branched with a single β-dgalactose at C-6 at about every nine residues in its main chain	
	PCP	6.710 × 10^4^	Ara, Gal, Glc, Xyl, GalA		[38]
	PCP	8.5 × 10^3^	Glc, Fru	→6)-β-D-Fru*f*-(2→,→1,6)-β-D-Fru*f*-(2→,→1)-β-D-Fru*f*-(2→,β-D-Fru*f*-(2→,and →6)-α-D-Gal*p*-(1→	[39]

**Table 3:** Concentration range of pharmacological activity of dietary polysaccharides from *Polygonatum sibiricum*.

Category	Component	Action Concentration (mg/kg/d)	Action Site	Pharmacological Activity	References
*Polygonatum sibiricum*	PSP	400	Knee osteoarthritis	PSP exerts anti-inflammatory and other effects in knee osteoarthritis by inhibiting the TLR2/NF-κ B signaling pathway.	[10]
*Polygonatum sibiricum Red* (leaves)	PSPs	200	Intestine	PsPs can increase the production of SCFAs by regulating the gut microbiota and have a positive prebiotic effect to regulate the gut.	[13]
*Polygonatum sibiricum*	PSP-H	100–400		Reduces insulin resistance index, increases OGTT and serum insulin levels, improves lipid metabolism, and enhances glucose metabolism in T2DM mice, thereby reducing blood glucose levels.	[46]
*Polygonatum sibiricum*	PSPW	50–200		PSPW may increase the phosphorylation levels of PI3K and Akt in the liver and decrease the phosphorylation levels of FoxO 1 and GSK 3 β by mediating the PI3K/AKT signaling pathway, thereby promoting improvement in the blood sugar in type 2 diabetes mice.	[47]
*Polygonatum sibiricum*	PSP		In vitro antioxidant	As the number of cooking cycles increases, the ability of PSP to scavenge DPPH free radicals gradually increases, and its IC 50 decreases from 3.30 ± 0.05 mg/mL to 0.11 ± 0.01 mg/mL at the ninth cooking cycle.	[49]
*Polygonatum sibiricum*	PSP	100–400		PSP plays a positive regulatory role in cellular and humoral immunity both in vivo and in vitro, and it can activate non-specific and specific immune responses (no immune-enhancing effect at low doses).	[51]
*Polygonatum sibiricum*	PSP	200	Tumor	PSP exerts an immune-enhancing effect against lung cancer by activating TLR4 receptors and downstream MAPK/NF-κ B signaling pathways.	[52]
*Polygonatum sibiricum*	PSP	100–400		PSP may prevent depressive-like behavior by reducing reactive oxygen species, hypercortisolism, and the inflammatory response and by decreasing synaptic and cellular damage.	[56]
*Polygonatum cyrtonema Hua*	PSP	400		PSP exerts antidepressant effects by regulating the oxidative stress-induced calpain 1-NLRP3 signaling axis.	[57]
*Polygonatum sibiricum*	PSP-N-c-1	100–400	Liver	The potential protective effect of PSP-N-c-1 on the liver may be related to the activation of the Nrf2-mediated signaling pathway and regulation of the TLR4-mediated NF-κ B signaling pathway, which can alleviate oxidative stress and the inflammatory response.	[58]

## Data Availability

No new data were created or analyzed in this study. Data sharing is not applicable to this article.
